# Safety of Short-course Weekly Rifapentine and Isoniazid (3HP) for TB Preventive Treatment at Conception and During First Trimester in Women With HIV: A Secondary Analysis of the WHIP_3_TB trial

**DOI:** 10.1093/cid/ciag315

**Published:** 2026-05-17

**Authors:** Violet Chihota, Zainab Waggie, Vicky Cardenas, Kathy Mngadi, Felex Ndebele, William Brumskine, Modulakgotla Sebe, Neil Martinson, Shu-Hua Wang, Getnet Yimer, Dinis Nguenha, Alberto L Garcia-Basteiro, Susan van den Hof, Alison D Grant, Salome Charalambous, Katherine L Fielding, Richard E Chaisson, Gavin Churchyard, for the WHIP3TB study team, Limakatso Lebina, Tumelo Moloantoa, Ebrahim Variava, Itumeleng Holele

**Affiliations:** Research Division, The Aurum Institute, Johannesburg, South Africa; School of Public Health, University of Witwatersrand, Johannesburg, South Africa; Department of Medicine, Vanderbilt University, Nashville, Tennessee, USA; Research Division, The Aurum Institute, Johannesburg, South Africa; Research Division, The Aurum Institute, Johannesburg, South Africa; Research Division, The Aurum Institute, Johannesburg, South Africa; Research Division, The Aurum Institute, Johannesburg, South Africa; Research Division, The Aurum Institute, Johannesburg, South Africa; Research Division, The Aurum Institute, Johannesburg, South Africa; Perinatal HIV Research Unit (PHRU), Faculty of Health Sciences, University of the Witwatersrand, Johannesburg, South Africa; Division of Infectious Disease, Department of Internal Medicine, The Ohio State University Global One Health Initiative, College of Medicine, Columbus, Ohio, USA; Center for Global Genomics and Health Equity, Department of Genetics, Perelman School of Medicine, University of Pennsylvania, Philadelphia, pennsylvania, USA; Centro de Investigação em Saúde de Manhiça (CISM), Maputo, Mozambique; Department of Global Health and Amsterdam Institute for Global Health and Development, Amsterdam University Medical Centers Location, University of Amsterdam, Amsterdam, The Netherlands; Centro de Investigação em Saúde de Manhiça (CISM), Maputo, Mozambique; ISGlobal, Hospital Clínic, Universitat de Barcelona, Barcelona, Spain; Centro de Investigación Biomédica en Red de Enfermedades Infecciosas (CIBERINFEC), Barcelona, Spain; Technical Division of TB elimination and Health Systems Innovations, KNCV Tuberculosis Foundation, The Hague, The Netherlands; National Institute of Public Health and the Environment, Centre for Infectious Disease Control, Bilthoven, The Netherlands; TB Centre, London School of Hygiene and Tropical Medicine, London, United Kingdom; Africa Health Research Institute, School of Laboratory Medicine & Medical Sciences, College of Health Sciences, University of KwaZulu-Natal, Durban, South Africa; Research Division, The Aurum Institute, Johannesburg, South Africa; School of Public Health, University of Witwatersrand, Johannesburg, South Africa; Department of Medicine, Vanderbilt University, Nashville, Tennessee, USA; TB Centre, London School of Hygiene and Tropical Medicine, London, United Kingdom; Center for TB Research, Johns Hopkins University School of Medicine, Baltimore, Maryland, USA; Research Division, The Aurum Institute, Johannesburg, South Africa; School of Public Health, University of Witwatersrand, Johannesburg, South Africa; Department of Medicine, Vanderbilt University, Nashville, Tennessee, USA

**Keywords:** HIV, TB, pregnancy, TB preventive treatment, rifapentine

## Abstract

**Background:**

The safety of a 3-month weekly rifapentine-isoniazid (3HP) for tuberculosis prevention in HIV-positive pregnant women on antiretroviral treatment is unknown. As part of the WHIP3TB trial, 3HP administered once or annually for 2 years was compared to 6 months of isoniazid. We report outcomes among women who became pregnant, with drug exposure in early pregnancy.

**Methods:**

Women were classified as exposed if conception occurred while taking study drug, and unexposed if conception occurred after the last dose. Outcomes included live birth, stillbirth, or abortion. A composite adverse pregnancy outcome was defined as any of the following: stillbirth, spontaneous abortion, low birth weight (<2500 g), preterm delivery (<37 weeks’ gestation), or major congenital anomalies. Outcomes were compared between exposed and unexposed groups.

**Results:**

Among 2207 women aged 15–49 years enrolled between November 2016 and November 2017, 236 pregnancies occurred in 222 participants. Fifty-seven (24%) were exposed to study drug (51 in 3HP arm, 6 in 6 months of isoniazid arm) and 179 were unexposed. Median age was 32 years, median antiretroviral treatment duration 2.9 years (interquartile range; 1.2–4.9 years) and median CD4 count 522 cells/L; [interquartile range, 345–684). Of 219 pregnancies with known outcomes, 162 (74%) were live births, 51 (23%) abortions, and 6 (3%) stillbirths. Spontaneous abortions occurred at similar frequencies among exposed (12% [7/57]) and unexposed (14% [22/162]) groups with comparable rates of composite adverse outcomes (33% [19/57] vs 35% [56/162]).

**Conclusions:**

Spontaneous abortion and composite adverse pregnancy outcomes following first-trimester exposure to 3HP were comparable between exposed and unexposed groups, supporting the safety of 3HP in pregnancy.

## Teaser

The Weekly High Dose Isoniazid and Rifapentine [P] Periodic Prophylaxis fo TB trial (WHIP_3_TB) trial found that weekly rifapentine-isoniazid for tuberculosis prevention in HIV-positive pregnant women showed no increased risk of adverse pregnancy outcomes, supporting its safety and potential use during pregnancy in high-burden settings.

Tuberculosis (TB) and HIV disproportionately affect women of reproductive age, with TB incidence peaking between 15 and 45 years [1–3]. Pregnant and postpartum women face a substantial risk of TB [4–6]. A recent review estimates that TB risk is 34% higher during pregnancy and 91% higher postpartum among HIV-negative women, and nearly 6 times higher during pregnancy and over 3-fold higher postpartum among women with HIV [7]. In 2023, 239 500 pregnant and 97 600 postpartum women developed TB globally, with HIV contributing to more than 20% of cases in pregnancy [7]. TB is associated with severe maternal complications, mortality, adverse pregnancy outcomes such as preterm birth and low birth weight, and heightened infant morbidity and mortality. Women are at increasingly diagnosed with TB within 90 days postpartum [4, 5, 8], although the timing of infection acquisition remains unclear. Treatment of TB infection in addition to antiretroviral treatment (ART) is recommended in people with HIV, including during pregnancy [9].

Isoniazid preventive treatment (IPT) has long been recommended during pregnancy, but data on its association with pregnancy outcomes are mixed [10]. Randomized controlled trials and secondary analyses show that initiating IPT during pregnancy increases adverse outcomes without added TB prevention benefit. The TB Ante vs. Postpartum PRevention with INH in HIV Seropositive mothers and their Expose Infants trial reported increased stillbirth, spontaneous abortion, low birth weight, and preterm delivery with antenatal versus postpartum IPT [11, 12]. Tshepiso cohort findings suggest fewer adverse outcomes when IPT is started later in pregnancy, with no harmful association in first trimester [13]. Conversely, Brief Rifapentine-Isoniazid Evaluation for TB prevention (BRIEF) TB, showed nearly doubled fetal demise risk with first trimester IPT [14]. In contrast, programmatic data from South Africa showed IPT during the second and third trimester reduced poor pregnancy outcomes in women with HIV [15]. As of January 2025, South Africa recommends antenatal IPT only for HIV-positive women with severe immunodeficiency.

Recent advances in TB preventive treatment (TPT) have introduced shorter regimens, notably 1HP (daily isoniazid and rifapentine for 1 month) and 3HP (weekly for 3 months), with better safety profiles and higher completion rates [16–20]. However, limited safety data on 3HP, in pregnant women with HIV, especially during early gestation restricts its use. International Maternal Pediatric Adolescent AIDS Clinical Trials Network 2001 reported adequate drug exposures and no major concerns, though small sample size limits interpretation [21]. Currently, the World Health Organization recommends only 6 months of daily isoniazid (6H) or 3 months of daily isoniazid and rifampicin during pregnancy [22]. Evidence on rifapentine-based TPT in pregnancy comes from secondary analyses: a review of the Three Months of Weekly Rifapentine and Isoniazid for M. Tuberculosis Infection (PREVENT TB) and Study 33: Adherence to Latent Tuberculosis Infection Treatment 3HP SAT Versus 3HO DOT (iAdhere) trials found no unexpected patterns of fetal loss or congenital anomalies among inadvertent 3HP/9H exposures, though numbers were small [23]. The study evaluating the safety and tolerability of one month daily and three months weekly isoniazid and rifapentine with pharmacokinetics of dolutegravir in pregnant people with HIV (DOLPHIN Moms) study in pregnant women with HIV suggests good tolerability for 3HP and 1HP started at 20–34 weeks, although coadministration of dolutegravir with 1HP may require double dosing [24, 25]. Collectively, data suggest differing safety profiles between early and later pregnancy, with sparse evidence for periconception and first-trimester rifapentine exposure.

The Evaluation of the Effect of 3HP vs Periodic 3HP vs 6H in HIV-Positive Individuals (WHIP_3_TB trial (NCT02980016), a pragmatic individually randomized trial, compared 3HP versus 6H in HIV-positive people, demonstrating high completion and good safety for 3HP [26]. At the time of study planning, due to lack of safety data on rifapentine during pregnancy, potential participants who were pregnant at enrollment were excluded. Although participants were counselled to avoid pregnancy while taking study drug, some participants conceived while taking study drug. The primary focus of the analysis is to assess the safety of 3HP among HIV-positive women who conceived during the WHIP_3_TB study, with 6H data presented for completeness and contextual purposes only.

## METHODS

### *Study* D*esign*

The parent study enrolled people with HIV, aged 2 years or older, receiving ART from health facilities in South Africa (5 sites), Ethiopia (2 sites), and Mozambique (1 site). The investigators’ institutional review boards approved the protocol. All participants or guardians gave written informed consent or witnessed verbal consent or assent as appropriate. Participants were randomized in a 9:9:2 ratio to receive either annual 3HP (900 mg of high-dose rifapentine and 900 mg of isoniazid taken weekly for 3 months and repeated annually) or the same regimen given once (both subsequently referred to as 3HP) versus 300 mg of isoniazid given daily for 6 months (6H) [26]. Randomization was stratified by country, using different block sizes. Pyridoxine (initially 25 mg per isoniazid dose, increased to 50 or 100 mg with each dose of isoniazid if peripheral neuropathy symptoms developed) was concurrently prescribed with the study drug. Participants in the 3HP groups and the 6H group were followed for 24 and 12 months, respectively. Part A of the trial compared treatment completion in the 3HP (combined rifapentine–isoniazid groups) versus the 6H arm over the first 12 months. Part B of the trial compared the effectiveness of rifapentine and isoniazid for preventing tuberculosis when given annually over 24 months versus once [26]. Urine pregnancy tests were performed at enrollment and in females who did not have a pregnancy test at baseline; those who were pregnant, breastfeeding, or of childbearing age and unwilling or unable to use contraception were excluded from the trial.

This analysis is restricted to participants of childbearing potential (15–49 years at enrollment) who became pregnant while in follow-up.

### Study Procedures for Participants of Childbearing Potential and Pregnancy

Women of childbearing potential in the 3HP arms were advised to use a secondary barrier method to prevent pregnancy during the 3 months they were on the study regimen. Urine pregnancy tests were performed at the month 12 and 24 visits in all women of childbearing potential. Following a positive pregnancy test, the last menstrual period (LMP) and estimated date of delivery (EDD) were ascertained.

Women who became pregnant while on the study drug were managed depending on their study arm: those assigned to either 3HP arms were discontinued from taking rifapentine, counselled about the risks and benefits of isoniazid, and provided with the option to switch to the 6H regimen (with consent). Pregnant women assigned to the 6H arm were counselled about the risks and benefits of isoniazid and given the option to continue treatment (with consent), or to stop. In all cases of pregnancy, women were encouraged to continue the study and complete the evaluations per the schedule of events. Given pregnancy testing was conducted at enrollment and at the initiation of second round of 3HP, and the isoniazid and rifapentine regimen was for 3 months, 3HP drug exposure was only possible in the first trimester of pregnancy.

Pregnancy outcomes were recorded from the “Road to Health Card” in South Africa, and the hospital notes in Mozambique and Ethiopia. The Road to Health Card is a record given at birth to monitor a child's growth, immunizations, and key health milestones up to age 5 years. These data were recorded as soon as the pregnancy outcome became known to the study staff. The time of recording these data varied, depending on the participant's study appointment date or how long it took to establish contact with the participant for their pregnancy outcome. The outcome and adverse events (AEs) for the participant and the infant were recorded on the AE case report form. While receiving treatment, participants were screened at follow-up visits for study-defined AEs including hepatotoxicity, peripheral neuropathy, psychosis, seizures, flulike reactions, and hypersensitivity reactions. Severity of AEs was graded according to the Division of AIDS toxicity table [27].

### Definitions

The study drug exposure period was defined as the interval between the first and last dose received. In the annual 3HP arm, a second exposure period corresponded to year 2 dosing. Pregnancies were categorized as exposed to 3HP or 6H or unexposed based on the estimated date of conception (EDC).

The EDC was derived using the LMP, EDD, or the date of the live birth (see Supplementary Figure 1 and Supplementary Figure 2). For live births, we used the infant's gestational age or assumed an average pregnancy length of 266 days if not known. Depending on data availability, up to 3 EDCs were calculated: LMP plus 14 days; EDD minus gestational age (or 266 days); and among live births the date of live birth minus 266 days. If at least 1 of the EDCs was within the study drug exposure period the pregnancy was classified as exposed to the relevant study drugs; and if all available EDCs were outside the study drug exposure period, then the pregnancy was classified as unexposed.

The pregnancy outcomes were documented as a live birth (including birth weight and gestational age), stillbirth, or an abortion (spontaneous, planned, and therapeutic). Abortion was defined as spontaneous (miscarriage) if the pregnancy ended naturally before 20 weeks, planned if the pregnant person chose to terminate the pregnancy through medical or surgical means, and therapeutic if abortion was performed due to medical necessity either because the mother's health was at risk or the pregnancy was not viable. In addition, pregnancy adverse outcomes were classified as maternal or perinatal outcomes. Adverse maternal outcomes included death during pregnancy or within 42 days postpartum, serious conditions occurring within 42 days postpartum or any condition requiring hospitalization. Perinatal deaths were defined as fetal deaths occurring after 26 completed weeks of pregnancy or deaths among live-born children up to 7 completed days of life. We defined a composite adverse pregnancy outcome as either stillbirth, spontaneous abortion, low birth weight (<2500 g), preterm delivery (<37 weeks’ gestation), or major congenital anomalies [14], defined using the standard Centers for Disease Control and Prevention structural criteria [28]. A composite severe adverse pregnancy outcome as either stillbirth, spontaneous abortion, very low birth weight (<1500 g), preterm delivery (<32 weeks’ gestation), or major congenital anomalies [11, 29]. We selected <32 weeks as the threshold for severe preterm delivery because births before 32 weeks represent the subgroup at highest risk of morbidity and mortality and is consistent with the standard World Health Organization categorization [29].

### *Statistical* A*nalysis*

All analyses were conducted in Stata version 18 (StataCorp, College Station, TX, USA). Among pregnant women, pregnancy outcomes were compared by study drug exposure and categorized as: “3HP,” “6H,” or “unexposed.” For the composite adverse pregnancy outcome, we reported risk ratios and risk differences and associated 95% confidence intervals (CIs) for 3HP and 6H drug exposure each, versus unexposed, using robust standard errors to control for multiple pregnancies. Components of the composite outcome are reported separately. All serious AEs that were not related to the pregnancy are reported by study arm. The number of pregnant women on 3HP at the time of their pregnancy test who were switched to 6H or stopped treatment, and on 6H at the time of their pregnancy test who stopped treatment was summarized.

## RESULTS

Of the 4014 individuals contributing to the “part A” primary outcome of the trial from November 2016 to November 2017, 2207 (55%) individuals were of childbearing potential and contributed to subsequent analyses; 1968 in the 3HP (986 single round, 982 in annually for 2 years) arm and 239 in the 6H arm (Supplementary Figure 3, Table 1). The median age was 40 years (interquartile range [IQR] 33–46 years), the median time on ART was 4.2 years (IQR 2.2–6.7 years), and 1474 (67%), 378 (17%) and 355 (16%) were enrolled in South Africa, Ethiopia, and Mozambique, respectively (Table 1).

**Table 1.** Demographics (All Measured at Enrollment into the Trial) by Arm for All Participants of Child-bearing Potential (n = 2207)

		Overall	3HP or p3HP	6H
Number of participants		2207	1968	239
Age, years:	Median [IQR]	40 (33–46)	40 (33–46)	39 (33–44)
Marital status	Single	1100 (47.8%)	1094 (47.8%)	126 (47.4%)
	Married/cohabiting	754 (32.9)	761 (33.3%)	80 (30.1%)
	Other	353 (19.3)	432 (18.9)	60 (22.6%)
Country of enrollment	South Africa	1474 (66.8%)	1318 (67.0)	156 (65.3)
	Ethiopia	378 (17.1%)	330 (16.8)	48 (20.1)
	Mozambique	355 (16.1%)	320 (16.3)	35 (14.6)
Years on ART	median [IQR]	4.2 (2.2–6.7)	4.2 (2.2–6.7)	4.0 (2.2–6.9)

Abbreviations: ART, antiretroviral therapy; IQR, interquartile range.

During the follow-up period, there were a total of 236 pregnancies in 222 participants. Among participants randomized to the 3HP arms, 11% (210/1968) had at least 1 pregnancy (196 having 1 pregnancy and 14 having 2 pregnancies) over the 24-month follow-up, and of those randomized to the 6H arm, 5% (12/239) participants had 1 pregnancy over the 12-month follow-up period.

Of the 236 pregnancies, most (179; 76%) were classified as unexposed to study drug; 51 and 6 were categorized as being exposed to 3HP or 6H, respectively (Table 2). Of the 14 participants who had 2 pregnancies while enrolled in the study, 3 (21%) had their first pregnancy exposed to 3HP and their second pregnancy unexposed, 2 (14%) had both pregnancies exposed to 3HP, 1 (7%) had their first pregnancy exposed to 3HP and the second exposed to 6H (participant had switched to 6H at the time of their first pregnancy), 1 (7%) had first pregnancy unexposed and second pregnancy exposed to 3HP, and 7 (50%) participants had both pregnancies unexposed. Among 51 participants exposed to 3HP, the median number of doses taken was 6 (IQR 4–8; range 1–12). Participants who conceived were a median age of 32 years (IQR 28–36), had median of 2 children (IQR 1–3), and the majority reported using barrier contraception (131/222) at enrollment. Their median time on ART was 2.9 years (IQR 1.2–4.9) and, their median CD4 count was 522 (IQR 345–684) cells/L. The median days on TPT to the first positive pregnancy test was 56 days (IQR 50–91) and 216 days (range 55–357) in the 3HP and 6H exposure groups, respectively. At study enrollment, 15/222 (7%) reported having hypertension; 13 of these were on antihypertensive medication. There were no self-reported diabetes, heart disease, or epilepsy, and 23/222 (10%) reported currently taking cotrimoxazole preventive therapy (see Supplementary Table 1).

**Table 2.** Summary (All Measured at Enrollment into the Trial, Except for Age) Overall and by Study Drug Exposure Group for First Pregnancies in Follow-up (n = 222)

	Overall	3HP Exposure	6H Exposure	Unexposed
First pregnancy (all pregnancies)	222 (236)	48 (51)	5 (6)	169 (179)
Country of enrollment:				
South Africa	157 (71%)	34 (71%)	5 (100%)	118 (70%)
Ethiopia	9 (4%)	1 (2%)	0	8 (5%)
Mozambique	56 (25%)	13 (27%)	0	43 (25%)
Age (years), median [IQR]	32 (28–36)	31 (27–37.5)	32 (25–34)^a^	32 (28–36)
Marital status:				
Single	127 (57%)	24 (50%)	3 (60%)	100 (59%)
Married/cohabiting	80 (36%)	23 (48%)	2 (40%)	55 (33%)
Other	15 (7%)	1 (2%)	0	14 (8%)
Level of education:				
<Grade 8	60 (27%)	13 (27%)	0	47 (28%)
Grade 8–11	104 (47%)	22 (46%)	3 (60%)	79 (47%)
≥Grade 12	58 (26%)	13 (27%)	2 (40%)	43 (25%)
#s who switched to 6H stopped Rx entirely			NA	NA
Parity, median [IQR]^b^	2 (1–3)	2 (2–3)	1 (0, 3)^a^	2 (1–3)
Smoker (yes)	10 (5%)	2 (4%)	0	8 (5%)
Previous TB (yes)	25 (11%)	8 (17%)	1 (20%)	16 (9%)
Type of contraception^c^:				
Oral	23	4	1	18
Injectable	53	10	2	41
Barrier	131	34	2	95
IUD	2	0	0	2
Implant	3	1	0	2
None	16	2	0	14
ART regimen—TDF + FTC/3TC + EFV or Atripla/Tribuss)	220 (99%)	47 (98%)	5 (100%)	168 (99%)
Time on ART (years), median [IQR]	2.9 (1.2–4.9)	3.6 (1.4–5.6)	2.4 (0.3–4.1)^a^	2.9 (1.2–4.7)
CD4 count (cells/L), median [IQR]^d^	522 (345–684)	619.5 (429–769)	225 (135–345)^a^	495 (329–658)
Days on TPT to first positive pregnancy test^e^, median [IQR]	NA	59 (50–101)	216 (55–357)^a^	NA

Abbreviations: ART, anti-retroviral therapy; IQR, interquartile range; TB, tuberculosis; TPT, TB preventive therapy.

^a^Data in parentheses are the range.

^b^Missing for n = 2 overall (n = 1 for 3HP exposure and n = 1 for unexposed).

^c^More than 1 contraceptive method may apply. Missing for n = 5 (all unexposed).

^d^Missing for n = 16 overall (n = 6 for 3HP exposure, n = 2 for 6H exposure and n = 8 for unexposed).

^e^Based on all pregnancies (time for pregnancies occurring in the second round of 3HP are measured from the first dispensing date in year 2. Positive pregnancy test is based on trial procedures, also a function of the frequency of follow-up visits.

In the 51 pregnancy episodes exposed to 3HP, 41% [21] resulted in a switch from 3HP to 6H and are summarized in the 3HP exposure group. The main reason for not switching was because 3HP had recently been completed (97%; 29/30); 1 participant had a spontaneous abortion 5 days before the visit when 3HP could have been stopped. None of the 6 participants who had a positive pregnancy test on 6H stopped their TPT.

Pregnancy outcome was unknown for 17/236 (7%) pregnancies, all of which were classified as unexposed to the study drug. Of the 219 known pregnancy outcomes in 207 women, 74% (162) were live births, 3% (6) were stillbirths, and 23% (51) were abortions (Table 3). Among the abortions, 57%, 27%, 8%, and 8% were spontaneous, planned, therapeutic, or unspecified, respectively. Planned abortions were more frequent in the 3HP exposure group versus the unexposed group (43% [6/14] versus 19% [7/36], respectively). Among the 162 live births, median gestational age was 39 weeks (IQR 38–40), median birth weight was 3 kg (IQR 2.7–3.3) and the mode of delivery was cesarean section in 20% (32). One baby died shortly after a cesarean section, with a birthweight of 1.14 kg, Apgar score at 5 minutes of 4, and Apgar score at 10 minutes of 6. The newborn was placed in an incubator at birth but died the same day. The event occurred in year 2 (second round of 3HP). The pregnancy outcome was recorded as a serious AE, and considered not related to rifapentine, isoniazid, and pyridoxine.

**Table 3.** **Pregnancy Outcomes Overall and by Study Drug Exposure Group (n** = **236)**

		Overall	3HP Exposure^a^	6H Exposure^a^	Unexposed^a^
Number of women		222	48^b^	6^b^	169
Number of pregnancies; outcome known		236; 219	51; 51	6; 6	179; 162
Outcome of pregnancy^c^	Live birth	162 (74%)	36 (71%)	4 (67%)	122 (75%)
	Stillbirth	6 (3%)	1 (2%)	1 (17%)	4 (2%)
	Abortion	51 (23%)	14 (27%)	1 (17)	36 (22%)
Among abortions	N	51	14	1	36
Abortion type	Spontaneous	29 (57%)	7 (50%)	0	22 (61%)
	Planned	14 (27%)	6 (43%)	1 (100%)	7 (19%)
	Therapeutic	4 (8%)	1 (7%)	0	3 (8%)
	Unspecified	4 (8%)	0 (0%)	0	4 (11%)
Among live births:	N	162	36	4	122
Gestational age, weeks	median (IQR)	39 (38–40)	39 (38–40)	38 (37–38)^d^	39 (37–40)
	n	157	35	4	118
Birth weight, kg	median (IQR)	3 (2.7–3.3)	2.8 (2.5–3.1)	3.2 (2.6–3.8)^d^	3.0 (2.7–3.3)
	n	159	35	4	120
Length, cm	median (IQR)	49 (48–51)	49 (47–50)	51 (46–53)^d^	49 (48–52)
	n	148	32	4	112
Head circumference,	median (IQR)	34 (33–36)	34 (32–35)	34 (33–35)^d^	34 (33–36)
cm	n	149	32	4	113
Apgar score at 5 min	median (IQR)	9 (9–10)	9 (8–10)	9 (9–10)^d^	9 (9–10)
	n	148	33	3	112
Apgar score at 10 min	median (IQR)	10 (10–10)	10 (9–10)	10 (10–10)	10 (10–10)
	n	116	26	3	87
Mode of delivery^e^	Normal delivery	127 (79%)	27 (75%)	4 (100%)	96 (79%)
	Ventoux/forceps	1 (1%)	09 (25%)	0	1 (1%)
	Cesarean section	32 (20%)		0	23 (19%)
Health status of child at birth^f^	Healthy	150 (99%)	31 (94%)	4 (100%)	115 (100%)
	Died	1 (1%)	1 (3%)	0	0
	Low birthweight	1 (%)	1 (3%)	0	0

^a^Of the 219 participants who have pregnancy outcome known, n = 12 had two pregnancies. Their exposure status is as follows: both pregnancies unexposed n = 6; both pregnancies 3HP exposed n = 2; first pregnancy exposed to 3HP and 2nd pregnancy unexposed n = 2; first pregnancy unexposed and 2nd pregnancy 3HP exposed n = 1; and first pregnancy exposed to 3HP and 2nd pregnancy exposed to 6H (participant had switched to 6H at the time of the first pregnancy).

^b^One trial participant had two pregnancies—1 exposed to 3HP and 1 exposed to 6H.

^c^n = 17 missing outcome (all from unexposed group).

^d^Data in parentheses indicate range.

^e^n = 2 missing type of delivery outcome (both from unexposed group).

^f^n = 10 missing child outcome (n = 3 from 3HP group and 7 from unexposed group).

Of the 236 pregnancies, outcomes were classified as serious AEs for 18 (8%) and unknown for 25 (11%) (2 in 3HP and 23 in the unexposed group). Eight percent (4/51), 17% (1/6), and 7% (13/179) of pregnancy outcomes were classified as serious AE, in the 3HP, 6H, and unexposed groups, respectively. These events were considered serious for the following reasons: 10 of 18 (55%) required inpatient hospitalization; 5 (27%) were stillbirths, 1 (6%) was a neonate death following 3HP exposure, 1 (6%) resulted in a maternal death, and 1 (6%) was considered life-threatening, both in the unexposed group (Table 4). The maternal death was reported as a uterine rupture and the pregnancy outcome was stillbirth.

**Table 4.** **Pregnancy Outcomes Considered Serious Adverse Events, Overall and by Study Drug Exposure Group (n** = **236)**

		Overall	3HP Exposure	6H Exposure	Unexposed
Number of pregnancies		236	51	6	179
Serious	Yes	18 (8%)	4 (8%)	1 (17%)	13 (7%)
	No	193	45	4	143
	Unknown	25	2		23
Pregnancy outcome	Live birth	5	2	0	3
	Still birth	5	1	1	3
	Abortion—spontaneous	3	0	0	3
	Abortion—therapeutic	4	1	0	3
	Abortion—unspecified	1	0	0	1
Reason for being serious (more than 1 reason possible)	Maternal death^a^	1	0	0	1
	Stillbirth or neonate death^b^	6	2	1	3
	Life threatening	1	0	0	1
	Required inpatient hospitalization	10	1	0	9
	Missing	2	1	0	1

^a^Maternal death uterine rupture.

^b^n = 5 are still births and n = 1 is neonate death (3HP arm).

Of 219 pregnancies with outcomes known, 75 (34%) satisfied the composite adverse pregnancy outcome; by exposure group, 35% (18/51) were exposed to 3HP, 17% (1/6) exposed to 6H and 35% (56/162) were unexposed to study drug. The risk ratios for adverse pregnancy outcome for 3HP and 6H exposure groups versus unexposed group were 1.02 (95% CI, 0.67–1.57) and 0.48 (0.08–2.93), respectively (Table 5). Of the components, spontaneous abortion and preterm delivery were similar in the 3HP exposure versus unexposed groups, and low birth weight was more common in the 3HP exposure versus unexposed groups (44% versus 30%, respectively). Of these 219 pregnancies, 16% (36/219) satisfied the composite severe adverse pregnancy outcome. Adverse pregnancy outcomes for first pregnancy are shown in Table 6.

**Table 5.** Adverse Pregnancy Outcomes for 219 Pregnancies in Analysis From 207 Women

	3HP Exposure	6H Exposure	Unexposed
Total number of pregnancies with known outcomes (N)	51	6	162
Composite adverse pregnancy outcome^a^: n	18	1	56
Components of the composite adverse pregnancy outcome^b^:			
Stillbirth n (%)	1 (5%)	1 (100%)	4 (7%)
Spontaneous abortion n (%)	7 (39%)	0	22 (39%)
Low birth weight (<2500 g) n (%)	8^c^ (44%)	0	17 (30%)
Preterm delivery (<37 weeks’ gestation n (%)	5 (28%)	0	15 (27%)
Major congenital anomalies n/N (%)	0	0	0
Composite adverse pregnancy outcome^a^: n/N (%)	18/51 (35%)	1/6 (17%)	56/162 (35%)
Risk ratio (95% CI)^d^	1.02 (0.67–1.57)	0.48 (0.08–2.93)	1
Risk difference (95% CI)^d^	0.7% (−14.3 to 15.7%)	−17.9% (−48.6 to 12.8%)	0
Composite severe adverse pregnancy outcome^a^: n/N (%)	9/51 (18%)	1/6 (17%)	26/162 (16%)

^a^n = 17 missing outcome (all from unexposed group).

^b^Percentage of those with an adverse pregnancy outcome. More than 1 reason can apply.

^c^n = 1 (birthweight 1.14 kg, gestational weeks 30) died on the same day as the live birth.

^d^Robust standard errors used to model repeated outcomes (women with >1 pregnancy).

**Table 6.** **Adverse Pregnancy Outcomes for First Pregnancy (n** = **207)**

	3HP Exposure	6H Exposure	Unexposed
Total number of pregnancies with known outcomes (N)	48	5	154
Composite adverse pregnancy outcome^a^: n	17	1	51
Components of the composite adverse pregnancy outcome^b^:			
Stillbirth, n (%)	1 (6%)	1 (100%)	4 (8%)
Spontaneous abortion, n (%)	7 (41%)	0	20 (39%)
Low birth weight (<2500 g), n (%)	7^c^ (41%)	0	16 (31%)
Preterm delivery (<37 weeks’ gestation), n (%)	5 (29%)	0	13 (25%)
Major congenital anomalies, n/N (%)	0	0	0
Composite adverse pregnancy outcome^a^: n/N (%)	17/48 (35%)	1/5 (20%)	51/154 (33%)
Risk difference (95% CI)	0.2% (−13.1 to 17.7%)	−13.1% (−49.0 to 22.7%)	0
Composite severe adverse pregnancy outcome^a^: n/N (%)	9/48 (19%)	1/5 (20%)	24/154 (16%)

^a^n=17 missing outcome (all from unexposed group).^b^of those with an adverse pregnancy outcome. More than one reason can apply.^c^n=1 (birthweight 1.14kg, gestational weeks 30) died on the same day as the live birth.

There was only 1 serious AE unrelated to pregnancy. The participant enrolled at the South Africa site (aged 34 years and on ART for 2.4 years) was randomized to 6H and developed drug-induced liver injury. The event occurred on 11 October 2017, 56 days after starting 6H. Laboratory results from 10 October showed aspartate transaminase 115 U/L and alanine transaminase 201 IU/L. On 11 October, results from a separate sample indicated aspartate transaminase at >3 times the upper limit of normal (the exact value was not recorded) and alanine transaminase, 205 IU/L (>5× upper limit of normal). The participant subsequently had a planned abortion, unrelated to the serious AE, on 18 October 2017 (63 days after starting 6H).

## DISCUSSION

In this cohort of 2553 participants of childbearing potential with HIV enrolled in a randomized TPT trial, there were a total of 236 pregnancies, from 222 participants, with one quarter exposed to 3HP or 6H. Because pregnancy was an exclusion criterion and 3HP was administered over 12 weeks, all 3HP exposures occurred around conception and in the first trimester; thus, our findings reflect early pregnancy exposure only. We found no difference in risk of the composite adverse pregnancy outcome in pregnant women exposed to 3HP or 6H compared to unexposed.

Notably, nearly 1 in 10 women became pregnant despite contraceptive counselling and consenting to pregnancy avoidance, underscoring persistent unintended pregnancy risk in high-burden settings and the need to evaluate TPT safety in early pregnancy, where inadvertent exposure is common.

Other studies evaluating the safety of 3HP in pregnancy have similarly not identified increased adverse outcomes with 3HP exposure [21, 23]. In our analysis, spontaneous and overall abortion rates were similar across exposure groups, consistent with PREVENT TB and iADHERE trial findings [23]. However, these findings contrast with BRIEF TB, which reported nearly doubled fetal demise risk with first-trimester daily IPT initiation [14]; differences in exposure duration/timing and baseline obstetric risk may explain this, underscoring the importance of regimen, duration, and gestational timing when assessing TPT safety in pregnancy

Population-based estimates from sub-Saharan Africa suggest low detected rates of pregnancy loss, though under-ascertainment is likely [30]. Our monthly follow-up may have captured early losses more completely. Within this context, similar spontaneous abortion frequencies across exposure groups suggest early 3HP exposure is unlikely to increase risk.

Our findings should also be interpreted within the broader context that women with HIV have a higher baseline risk of adverse pregnancy outcomes, even without TPT. In sub-Saharan Africa, HIV infection is associated with increased risks of preterm birth and low birth weight independent of ART status with modelling studies estimating >2 million adverse perinatal outcomes attributable to HIV and ART between 1990 and 2020, particularly among women on preconception ART [31, 32]. Against this elevated baseline, our finding that 3HP exposure showed no increase in spontaneous abortion or other adverse outcomes is reassuring, supporting 3HP's safety around conception. Findings from Rifapentine-containing Tuberculosis Treatment Shortening Regimens for Pulmonary TB Study 31/ACTG A5349 similarly demonstrated that brief first-trimester exposure is not associated with excess fetal loss or congenital anomalies, though primarily among HIV-negative women. Despite variable background rates, our findings add needed evidence for pregnant women with HIV.

In our study, pregnancy outcomes classified as serious AEs were uncommon, reported in 9% of all pregnancies, with a higher percentage in pregnancies exposed to 6H, though numbers were small. Most were considered serious because they required inpatient hospitalization and most were in the unexposed group. There was 1 maternal death due to uterine rupture in an individual in the unexposed group. In our study, no major congenital anomalies were reported in the 3HP arm. This is consistent with findings from 31 pregnant women given 3HP in the PREVENT and iADHERE trials [23]. In these trials, although no unexpected congenital anomalies were reported, 2 anomalies were reported among 56 women in the 9H arm.

Our study has limitations. It was not designed to assess TPT safety during pregnancy, and exposures occurred only in early pregnancy; therefore, our results cannot inform safety in later gestation. Pregnancy outcomes derived from routine data may be incomplete, and infants’ assessments were not systematic. Some outcome data were missing among unexposed participants. To determine drug exposure, we used multiple methods to estimate EDC, with some discrepancies between dates. We used a conservative approach to allocating pregnancies to treatment exposure, based on dates falling within the exposure period. This could result in misclassification whereby some pregnancies defined to be exposed to study treatment were in fact not, potentially masking any relationship to composite adverse pregnancy outcome. Given similar composite adverse pregnancy outcome frequencies in the exposed and unexposed groups we do not consider this a major limitation. Because data were primarily collected in South Africa, differences in health-system capacity and antenatal care may limit generalizability to other high-burden settings, though no biological differences in drug effects or pregnancy outcomes are expected. Finally, variability in exposure timing and duration relative to conception contributes to understanding short-course exposure around conception and early embryogenesis, complementing studies of later in pregnancy exposure.

## CONCLUSION

Because pregnancy was an exclusion criterion, all 3HP exposure in this study occurred inadvertently in early pregnancy. Spontaneous abortions and composite outcomes were similar in the 3HP exposed and unexposed groups with no unexpected fetal loss patterns and or major congenital anomalies.

## Supplementary Material

ciag315_Supplementary_Data

## Supplementary Data

Supplementary materials are available at *Clinical Infectious Diseases* online. Consisting of data provided by the authors to benefit the reader, the posted materials are not copyedited and are the sole responsibility of the authors, so questions or comments should be addressed to the corresponding author.
