# Supplementary material for: Safety of Short-course Weekly Rifapentine and Isoniazid (3HP) for TB Preventive Treatment at Conception and During First Trimester in Women With HIV: A Secondary Analysis of the WHIP3TB trial
Source: Clin Infect Dis. 2026 May 17;83(3):e550–8. doi: 10.1093/cid/ciag315 (PMC13644861; doi:10.1093/cid/ciag315)
Supplement: Supplementary file 1 [file ciag315_supplementary_data.docx]

**Supplemental data for : Chihota et al. Safety of short-course weekly rifapentine and isoniazid (3HP) for TB preventive treatment in pregnant HIV infected women**

**Figure S1: Differences in expected date of conception, by method – restricted to live births (n=160)**

The plot shows difference in days for each estimated date of conception (EDC) versus the EDC calculated from the actual delivery date, restricted to live births. Dashed vertical lines show differences in EDCs of ±14 days

Footnote: EDC estimated date of conception; LMP last menstrual period, EDD estimated date of delivery, gestage gestational age

For EDC using LMP (EDC_LMP_), only 50% (79/158) of the dates are within ±14 days of EDC calculated from the actual delivery date (EDC_ADD_), 11% (17/158) have EDC_LMP_ earlier (<-14 days) than EDC_ADD_ and 39% (62/158) have EDC_LMP_ later than EDC_ADD_ (>14 days).

Results are similar for EDC calculated from the estimated delivery date (EDC_EDD_); 52% (84/160) of the EDC_EDD_ are within ±14 days EDC_ADD_, 12% (19/160) have EDC_EDD_ earlier (<-14 days) than EDC_ADD_ and 36% (57/160) have EDC_EDD_ later than EDC_ADD_ (>14 days).

EDC using gestational age (EDC_ga_) and EDC_ADD_ are more consistent; 87% (135/156) of the dates are within ±14 days of each other, 7% (11/156) have EDC_ga_ earlier (<-14 days) than EDC_ADD_ and 6% (10/156) have EDC_ga_ later than EDC_ADD_ (>14 days).

**Figure S2: Differences in expected date of conception, by method – restricted to non-live births (n=73)**

The plot shows difference in days for the estimated date of conception (EDC) using last menstrual period and the EDC calculated from the estimated delivery date, restricted to non-live births. Dashed vertical lines show differences in EDCs of ±14 days

 Footnote: EDC estimated date of conception; LMP last menstrual period, EDD estimated date of delivery,

For EDC using LMP (EDC_LMP_), 82% (60/73) of the dates are within ±14 days of EDC calculated from the estimated delivery date (EDC_EDD_), 4% (3/73) have EDC_LMP_ earlier (<-14 days) than EDC_EDD_ and 14% (10/73) have EDC_LMP_ later than EDC_EDD_ (>14 days).

**Figure S3: Study Flow for Pregnancy Exposure Groups**

**^1^ Churchyard et al. Ann Int Med 2021; 3HP isoniazid and rifapentine (weekly for three months); 6H isoniazid (daily for six months)**

**Table S1: other health conditions self-reported at study enrolment**

|  | **Overall** | **3HP exposure** | **6H exposure** | **Unexposed** |
| --- | --- | --- | --- | --- |
| **First pregnancy** | **222** | **55** | **6** | **161** |
| **High blood pressure/hypertension**  **On treatment** | **15 (6.7%)**  **13/15** | **8 (14.6%)**  **8/8** | **1 (16.7%)**  **1/1** | **6 (3.7%)**  **5/6** |
| **Diabetes** | **0** | **0** | **0** | **0** |
| **Heart disease** | **0** | **0** | **0** | **0** |
| **Epilepsy** | **0** | **0** | **0** | **0** |
| **On CPT** | **23 (10.4%)** | **5 (9.1%)** | **0** | **18 (11.2%)** |

CPT Cotrimoxazole Preventive Therapy/Bactrim

**Alt Text**

**Figure S1:**
Scatter plot showing differences in days between estimated date of conception derived from last menstrual period, estimated delivery date, or gestational age compared with estimated date of conception calculated from the actual delivery date among live births (n=160). Vertical dashed lines indicate differences of ±14 days.

**Figure S2:**
Scatter plot showing differences in days between estimated date of conception derived from last menstrual period and estimated delivery date among non‑live births (n=73). Vertical dashed lines indicate differences of ±14 days.

**Figure S3:**
Flow diagram showing participant inclusion and classification into pregnancy exposure groups, including weekly rifapentine and isoniazid for three months (3HP), daily isoniazid for six months (6H), and unexposed groups, with participant numbers at each stage.
